# Personalizing Care Through Robotic Assistance and Clinical Supervision

**DOI:** 10.3389/frobt.2022.883814

**Published:** 2022-07-12

**Authors:** Alessandra Sorrentino, Laura Fiorini, Gianmaria Mancioppi, Filippo Cavallo, Alessandro Umbrico, Amedeo Cesta, Andrea Orlandini

**Affiliations:** ^1^ Scuola Superiore Sant’Anna, Pisa, Italy; ^2^ Department of Industrial Engineering, University of Florence, Florence, Italy; ^3^ CNR–Institute of Cognitive Sciences and Technologies (CNR-ISTC), Rome, Italy

**Keywords:** socially assistive robot (SAR), knowledge representation and reasoning (KRR), automated planning (AP), user modeling (UM), human–robot interaction (HRI)

## Abstract

By 2030, the World Health Organization (WHO) foresees a worldwide workforce shortfall of healthcare professionals, with dramatic consequences for patients, economies, and communities. Research in assistive robotics has experienced an increasing attention during the last decade demonstrating its utility in the realization of intelligent robotic solutions for healthcare and social assistance, also to compensate for such workforce shortages. Nevertheless, a challenge for effective assistive robots is dealing with a high variety of situations and *contextualizing* their interactions according to living contexts and habits (or preferences) of assisted people. This study presents a novel cognitive system for assistive robots that rely on artificial intelligence (AI) representation and reasoning features/services to support decision-making processes of healthcare assistants. We proposed an original integration of AI-based features, that is, *knowledge representation and reasoning* and *automated planning* to 1) define a human-in-the-loop continuous assistance procedure that helps clinicians in evaluating and managing patients and; 2) to dynamically adapt robot behaviors to the specific needs and interaction abilities of patients. The system is deployed in a realistic assistive scenario to demonstrate its feasibility to support a clinician taking care of several patients with different conditions and needs.

## 1 Introduction

By 2030, the World Health Organization (WHO) foresees a worldwide workforce shortfall of about 18 million healthcare professionals, with dramatic consequences for patients, economies, and communities (Liu et al., 2017). The development of ICT-based integrated care solutions offers a variety of possible solutions to address this issue. Research in assistive robotics has experienced an increasing attention during the last decade aiming at the realization of intelligent robotic solutions for healthcare and social assistance, also to compensate for such workforce shortages. Also, the potential impact of healthcare and assistive robots is also witnessed by their deployments to deal with the COVID19 pandemic (Murphy et al., 2022). Remarkable results have been achieved integrating social robots in realistic assistive scenarios with human users (see e.g., (Cavallo et al., 2018; Angelini et al., 2019; D’Onofrio et al., 2016; Bertolini et al., 2016; Coradeschi et al., 2013)), also including the case of assistance and monitoring of impaired and frail people (see, e.g., (Casey et al., 2016; Fiorini et al., 2017; Mancioppi et al., 2019)). Assistive robots can be then used to support healthcare professionals in their activities augmenting their capacities and strength in dealing with a wide number of patients. Moreover, human–robot interaction (HRI) is now a very compelling field also used to better understand how humans perceive, interact with, or accept these machines in social contexts (Wykowska et al., 2016). Several studies investigated the relationships between user needs and assistive robot features when deployed inside integrated care solutions for older adults living alone in their homes (see, e.g., (Cesta et al., 2018; Cortellessa et al., 2021)). A crucial requirement for effective assistive robotic systems is their ability to deal with a high variety of situations and *contextualize* their interactions according to living contexts and habits (or preferences) of assisted people (Rossi et al., 2017; Bruno et al., 2019; Umbrico et al., 2020c). A key current challenge consists in realizing advanced control systems endowing assistive robots with a rich portfolio of high-level cognitive and interaction capabilities (Nocentini et al., 2019; Fiorini et al., 2020a) to realize *personalized* and *adaptive* assistance (Tapus et al., 2007; Umbrico et al., 2020a; Andriella et al., 2022) and thus achieve a good level of *acceptance* (Rossi et al., 2017; Moro et al., 2018).

This study presents a cognitive system for assistive robots that rely on ontology-based representation and reasoning capabilities to support healthcare professionals and elderly users during assessment and therapy administration. More specifically, the presented approach pursues a human-in-the-loop methodology that leverages a “robot-based” user profiling and artificial intelligence (AI) representation and reasoning features/services to support decision-making processes of healthcare assistants. The objective is, on the one hand, to support healthcare professionals during patient assessment and therapy administration and, on the other hand, to provide assistive robots with *personalization* and *adaptability* features to support patients characterized by heterogeneous health-related needs. Taking inspiration from cognitive architecture research (Langley et al., 2009; Lieto et al., 2018; Kotseruba and Tsotsos, 2020), we proposed the integration of AI-based features, that is, *knowledge representation and reasoning* and *automated planning* to 1) define a human-in-the-loop process for continuous evaluation and treatment of patients and; 2) to dynamically adapt robot behaviors to the specific needs and interaction abilities of patients.

The system is deployed on a social assistive robot and validated in a realistic scenario. We showed how an assistive robot endowed with cognitive control features is able to autonomously contextualize its behavior and effectively support both patients and clinicians in the synthesis of personalized cognitive interventions. A key point stands in the *mutual assistance* between the clinician and the robot through a “mixed-initiative” work flow. The role of the clinician is essential to *refine* and *validate* decisions made by the robot. In turn, the robot supports the clinician in the *screening* and *monitoring* of patients as well as the *administration* of a therapy. In this regard, the main contribution of the work concerns the correlation between *standard* screening practices used by therapists with the internal user model used by the robot. This correlation allows a robot to correctly interpret health-related data about patients provided by therapists. In particular, it enables the *transfer* of knowledge from clinicians to robots and is thus crucial to synthesizing effective and personalized assistive behaviors.

A profiling procedure is performed through a robotic platform during the administration of the Mini-Mental State Examination (MMSE) to patients with suspected cognitive decline. As shown in Rossi et al. (2018) and Di Nuovo et al. (2019), the use of a robot guarantees test neutrality and attainable standardization for the administration of cognitive tests. Data about the quality of interaction are extracted to refine interaction modalities and thus shape robot behaviors when interacting with users. *User modeling* capabilities of the robot rely on an ontological reification of the *International Classification of Functioning, Disability and Health* ^1^ (ICF). The obtained ontological model defines a well-structured and general reference framework suitable to autonomously reason about the health status of a person and elicit fitting interaction parameters. Many works in the literature deal with user modeling and propose different frameworks, depending on the specific application needs (Lema ignan et al., 2010; Awaad et al., 2015; Tenorth and Beetz, 2015; Lemaignan et al., 2017; Porzel et al., 2020).

Concerning healthcare and assistive domains, user modeling is particularly crucial to support a *user-centered design* and realize effective assistive technologies (LeRouge et al., 2013). Other works have used the ICF framework as a reference to characterize cognitive and physical conditions of users. For example, the work (Kostavelis et al., 2019) introduced a novel robot-based assessment methodology of users’ skills is proposed in order to characterize the needed level of daily assistance. The work (Filippeschi et al., 2018) used the ICF to characterize cognitive and physical skills of users and accordingly represent the outcomes of the implemented robot-based assessment procedures. Similarly, the work (García-Betances et al., 2016) used ICF to represent needs and requirements of different types of users and support the user-centered design of ICT technologies. In particular, this work integrates an ontological model of ICF into the cognitive architecture ACT-R (Anderson et al., 1997) to simulate the behaviors of different types of user.

Nevertheless, the aforementioned works present a “rigid” and static representation as they usually do not rely on a well-structured ontological formalism to characterize *knowledge* about users (i.e., *user profiles*) in different situations. Related works usually do not integrate online reasoning mechanisms that allow assistive robots to *autonomously reason* about the specific needs of a user and autonomously (or partially autonomously) *decide* the kind of intervention that best fit such needs. Conversely, our approach pursues a highly flexible solution implementing the cognitive capabilities needed to *understand* health conditions of users and (autonomously) personalize assistance accordingly, under the supervision of a human expert.

## 2 Continuous Assessment and Monitoring

We aimed at leveraging the interaction capabilities of socially assistive robots to support clinicians in assessing and monitoring the cognitive state of patients. We envisage a multi-actor HRI approach in which an assistive robot can facilitate the interactions. In particular, we proposed a continuous assessment and monitoring procedure in which a robot supports a clinician by: 1) *proposing* a set of tests suitable for the specific needs of a patient, 2) *administering* the chosen tests, and 3) *monitoring* (and reasoning over) the performance of the patient. In this way, the employment of assistive robots can alleviate clinicians in some of their activities and, thus, support them in dealing with a larger number of patients. In addition, a robot can continuously and proactively stimulate patients by administrating suitable exercises and generally motivating the *participation* and the *adherence* to the therapy.

### 2.1 Mixed-Initiative Design of Cognitive Stimulation Therapy

**FIGURE 1:**
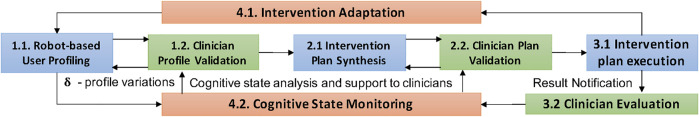
Workflow of the devised cognitive intervention mixing on robot and human in skills.

We envisage a novel cognitive intervention program where a robot constantly supports clinicians in evaluating/monitoring the cognitive state of a patient and in making decisions about the *intervention plan* to follow. The general structure is depicted in Figure 1. The process fosters a continuous “feedback loop” between the robot and the clinician. It interleaves patient–robot interactions (i.e., steps 1.1, 2.1, and 3.1 in Figure 1) with direct clinician validation and involvement (i.e., steps 1.2, 2.2, and 3.2 in Figure 1). The interleaving of steps performed by the two actors aimed at achieving a fruitful synergy combining the computational capabilities of the robot with the analytical capabilities of the clinician.

It is worth noticing that the clinician is constantly involved in the decisional process and maintains control over the decisions made by the robot, validating them. Each cycle consists of a number of human–robot interaction steps aiming at 1) profiling the health state of a person (steps 1.1 and 1.2), 2) defining an intervention plan suitable for the specific health needs of a patient (steps 2.1 and 2.2) and, 3) executing the plan by administrating exercises within a certain temporal horizon (e.g., a week or a month) and evaluating outcomes (steps 3.1 and 3.2).

The cyclic repetition of these phases allows a clinician to continuously monitor and assess the *evolving cognitive state* of a patient with the support of a robot. Two “feedback chains” are considered as shown in Figure 1. One feedback chain assesses and (if necessary) updates the profile of the user at the end of each cycle. In this way, it is possible to keep track of the outcomes of synthesized plans, keep track of changes in the health state of a person, and adapt the next cycle accordingly. Another feedback chain concerns the continuous construction of a dataset containing information about the evolution of user profiles and the related outcomes of the cognitive interventions. This information would, in particular, allow the clinician to analyze the evolution over time of the state of a user and thus make better decisions about next steps.

### 2.2 An AI-Based Cognitive Architecture

To implement the process of Figure 1, an assistive robot should be able to reason about the health state/conditions of a patient and autonomously make suitable decisions. In particular, a robot needs a number of properly designed and integrated *cognitive capabilities* in order to contextualize assistive behaviors and effectively support both patients and clinicians. Taking inspiration from cognitive architectures (Langley et al., 2009; Kotseruba and Tsotsos, 2020), we focus on the development and integration of AI-based technologies supporting *knowledge representation and reasoning* and *decision making and problem solving*. The integration of knowledge representation and reasoning with automated planning has been shown to be effective for the synthesis of flexible robot behaviors. They are particularly crucial to realize advanced (cognitive) controllers capable of (autonomously) *personalize* and *adapt* robot behaviors to the specific features of different application scenarios, for example, *service robots* (Awaad et al., 2015; Tenorth and Beetz, 2015; Porzel et al., 2020), *daily assistance* (Umbrico et al., 2020a; Cortellessa et al., 2021), and *manufacturing* (Borgo et al., 2019).

**FIGURE 2:**
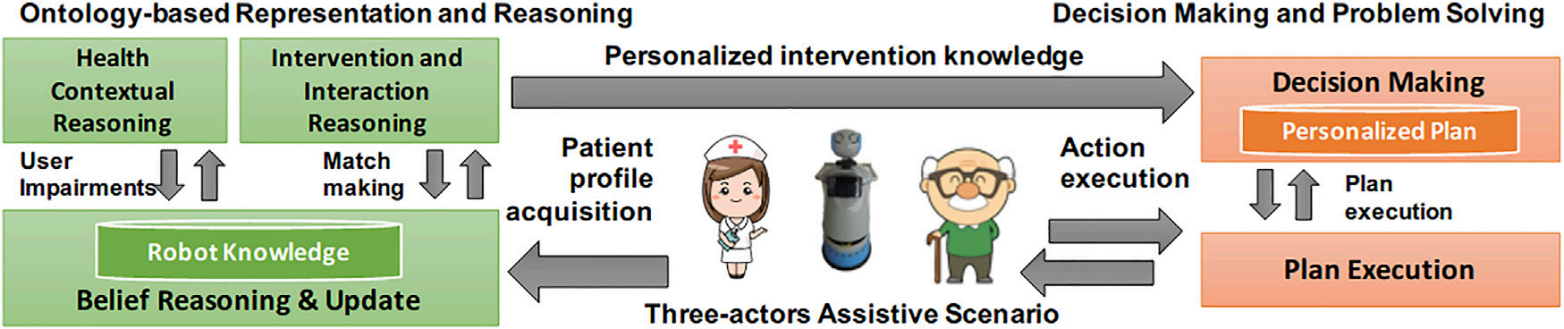
Overview of the AI-based cognitive architecture.

Figure 2 provides an overview of modules developed to support the considered capabilities and their integration within a “cognitive loop.” On the one hand, an *ontology-based representation and reasoning* module allows an assistive robot to internally represent cognitive and physical information about an assisted person and contextualize its interaction and intervention capabilities accordingly. Pursuing a foundational approach (Guarino, 1998), we defined a domain ontology based on the ICF classification to represent user profiles and reason on the health state of a person. It relies on DOLCE^2^ as a theoretical foundation, and was written in OWL (Antoniou and Harmelen, 2009) using Protégé^3^. The *robot knowledge* and related knowledge-reasoning modules have been developed in Java using the open-source library Apache Jena^4^. On the other hand, a *decision making and problem solving* module allows an assistive robot to synthesize and execute intervention *plans*, personalized according to the “recommendations” extracted from the robot knowledge. The synthesis and execution of such plans rely on PLATINUm (Umbrico et al., 2017), a timeline-based planning and execution framework deployed in assistive scenarios (Umbrico et al., 2020a) and concrete human–robot collaboration manufacturing scenarios (Pellegrinelli et al., 2017). The contribution of this work specifically focuses on the developed ontology-based representation and reasoning capabilities.

## 3 Ontology-Based Modeling of Health-Related Needs

A user profile should encapsulate a rich and heterogeneous set of information characterizing the general health state of a person. We proposed an ontological model of health needs based on the *International Classification of Functioning, Disability and Health* (ICF), defined by the World Health Organization (WHO) (World Health Organization, 2001). User profiles are thus represented on top of such ICF-based ontological models in order to provide an assistive robot with a complete characterization of patients’ needs.

### 3.1 Modeling Health-Related Knowledge About Patients

There are several factors that can be considered when modeling users. Different choices would support different robot behaviors and different levels/types of adaptation. Broadly speaking, the creation of a complete and effective user model is crucial to realize human–robot interactions characterized by adaptability, trust building, effective communication, and explainability (Tabrez et al., 2020). In the context of cognitive assessment with assistive robots, many works tend to focus on personality, emotions, and engagement as aspects of the user profile that the robot should take into account (Sorrentino et al., 2021). For example, Tapus et al. (2008) described a socially assisted robot therapist designed to monitor, assist, encourage, and socially interact with post-stroke users engaged in rehabilitation exercises. This work investigated the role of the robot’s personality (i.e., introvert–extrovert) in the therapy process taking into account the personality traits of a user. Similarly, (Rossi et al. (2018) investigated the influence of the user’s personality traits on the perception of the Pepper robot, administrating a cognitive test. Their results suggested that the usage of a robot in this context improved socialization among the participants. On the other hand, the works of Desideri et al. (2019) and (Pino et al. (2020) showed that the usage of a robotic platform for cognitive stimulation engaged more participants to the therapy. In the mentioned works, the influence of each aspect was mostly investigated offline and it was mostly related to the occurred quality of the interaction. In addition, the robotic platform was adopted as a medium for the administration of the clinical protocol, without providing any cues on how the information collected by the robot could be used for planning future interventions. The assumption behind this work is that the robot should be able to adapt its intervention, by focusing on the quality of the interaction, but also on the user cognitive profile.

Concerning our contribution, other works have used the ICF framework to characterize cognitive and physical conditions of users. The work (Kostavelis et al., 2019) introduced a novel robot-based assessment methodology of users’ skills to characterize the needed level of daily assistance. The work (Filippeschi et al., 2018) used ICF to characterize cognitive and physical skills of users and accordingly represent the outcomes of the implemented robot-based assessment procedures. Similarly, the work (García-Betances et al., 2016) used ICF to represent needs and requirements of different types of users and support a user-centered design of ICT technologies. This work integrates an ontological model of ICF into the cognitive architecture ACT-R (Anderson et al., 1997) to simulate the behaviors of different types of user. Nevertheless, these works present a “rigid” and static representation as they usually do not rely on a well-structured ontological formalism to dynamically contextualize *knowledge* about users (i.e., *user profiles*) in different situations. Such works usually do not integrate online reasoning mechanisms to allow assistive robots to *autonomously reason* about the specific needs of a user and autonomously (or partially autonomously) *decide* the kind of intervention that best fit such needs. Conversely, our approach pursues a highly flexible solution implementing the cognitive capabilities needed to *understand* health conditions of users and (autonomously) personalize assistance accordingly, under the supervision of a human expert.

### 3.2 ICF-Based Representation of User Profiles

The ICF classification aimed at organizing and documenting information on functioning and disability. It pursues the interpretation of functioning as a dynamic interaction among health conditions of a person, environmental factors, and personal factors. Each defined concept characterizes a specific aspect concerning the physical or cognitive functioning of a person. The level of functioning of each physical/cognitive aspect is represented by the following scale: 1) the value 0 denotes *no impairment*, 2) the value 1 denotes *soft impairment*, 3) the value 2 denotes *medium impairment*, 4) the value 3 denotes *serious impairment*, and 5) the value 4 denotes *full impairment*.

The ICF classification is organized into two parts. A part deals with *functioning and disabilities* while the other part deals with *contextual factors*. The former is further organized into the components *body functions* and *body structures* that are the ones considered in the design of the ontological model. The body is an integral part of human functioning and the bio-psychosocial model considers it in interaction with other components. Body functions are thus the physiological aspects of body systems, while structures are the anatomical support (e.g., sight is a function while the eye is a structure). Several ICF concepts describe the functioning of mental faculties and have been used to define user profiles. The concept OrientationFunctioning characterizes the functioning of general mental functions of known and ascertaining one’s relation to time, to place, to self, objects, and space. The concept AttentionFunctioning characterizes specific mental functions focusing on external stimulus or internal experience for the required period of time. The concept MemoryFunctioning characterizes specific mental functions of encoding, storing information, and retrieving it as needed.

Other ICF concepts have been instead used to characterize the *interaction capabilities* of a person and thus identify *interaction preferences* determining the way a robot should interact with a person while administrating exercises. The concept SeeingFunctioning models specific functions related to seeing the presence of light and sensing the form, the size, shape, and color of visual stimuli. The concept HearingFunctioning models sensory functions related to sensing the presence of sounds and discriminating the location, pitch, loudness, and quality of sounds.

## 4 Knowledge Reasoning for Personalization

Information gathered during the profiling phase and its representation based on ICF allow an assistive robot to autonomously reason about the intervention plan that “best fit” the specific needs of a person (e.g., *what* kind of cognitive exercise a person needs) and the way such actions should be executed (e.g., *how* a robot should interact with a person to effectively administrate cognitive exercises).

### 4.1 From Impairments to Intervention Actions

Following ICF classification, the ontological model defines a number of concepts that represent different FunctioningQuality of a person. As mentioned in Section 2.2, we rely on DOLCE as foundational ontology. Then, the ICF qualities are modeled as subclasses of DOLCE:Quality and are associated to entities of type DOLCE:Person. The concept Profile defines a descriptive context of the overall functioning qualities of a particular person. It represents the outcome of a profiling phase and consists of a number of Measurements. Each measurement associates the evaluation of a functioning quality to a *value* representing the assigned ICF score (i.e., the outcome of the evaluation). Knowledge-reasoning processes analyze such measurements (i.e., a user profile) to autonomously *infer* the physical or cognitive impairments characterizing the functioning state of a person. Eq. 1 in the following section shows a general inference rule used to detect such impairments.

$$\begin{array}{cc} \forall \text{x, y, w.}\exists \text{z.}(\text{Measurement}(\text{x})\wedge & \\ \text{measures}\left(\text{x, y}\right)\wedge & \\ \text{FunctioningQuality}\left(\text{y}\right)\wedge & \\ \text{hasOutcome}\left(\text{y, w}\right)\wedge & \\ \text{FunctioningRegion}\left(\text{w}\right)\wedge & \\ \text{greaterThan}\left(\text{hasICFscore}\left(\text{w}\right)\text{, 0}\right)\wedge & \\ \text{lowerThan}\left(\text{hasICFscore}\left(\text{w}\right)\text{, 5}\right)\to & \text{Impairment}\left(\text{z}\right)\wedge \\ & \text{concerns}\left(\text{z, y}\right)\wedge \\ & \text{satisfies}(\text{z, x})). \end{array}$$ (1)

In addition to this knowledge, the ontology characterizes *properties* of intervention plans. A robot can indeed be endowed with a number of “programs” implementing known tests suitable to evaluate/stimulate different functioning qualities, for example, the *Free and Cued Selective Reminding Test* for episodic long-term memory assessment or the *Trailing Making Test form A* for selective attention assessment.

Taking inspiration from some works in manufacturing that define the concept of function (Borgo and Leitão, 2004; Borgo et al., 2009), we characterized intervention actions of a robot in terms of their *effects* on the functioning qualities of a person. The defined semantics characterizes these “programs” according to the functioning qualities they address. For example, an interactive program implementing the *Free and Cued Selective Reminding Test* is classified as an intervention action whose effects can “improve” (i.e., *has positive effects on*) the functioning quality MemoryFunctioning. The obtained ontological model fosters an integrated representation of knowledge about the health state of a person and intervention capabilities of a robot. Knowledge processing mechanisms then use this integrated knowledge to infer a set of actions suited to address the inferred impairments of a patient (Umbrico et al., 2020b). For example, if MemoryFunctioning is inferred as *soft impairment* (value 1) and AttentionFunctioning as *no impairment* (value 0) only actions implementing the *Free and Cued Selective Reminding Test* (or other similar tests) are inferred as suitable to a patient.

### 4.2 Reasoning on Interaction Preferences

A Profile encapsulates a rich set of information that can be analyzed to infer interaction capabilities of a person and define robot interaction preferences accordingly. If the analysis of a profile infers a *medium impairment* of the quality HearingFunctioning then, the interactions between the patient and the robot should rely mainly on visual and textual messages rather than voice and audio. In case that audio interactions cannot be avoided (e.g., recorded audio instructions and recommendations or video conferences) it would be possible to properly set the sound level of the robot in order to help the assisted person as much as possible.

Knowledge-reasoning mechanisms thus infer also *how* intervention plans should be carried out by the robot in order to effectively interact with the considered patient. In this regard, we have defined four interaction parameters characterizing the execution of robot actions: 1) *sound level*, 2) *subtitle*, 3) *font size*, and 4) *explanation*. The *sound level* is an enumeration parameter with values \{ *none*, *regular*, *high*\} specifying the volume of audio communications and messages from the robot to the patient. Patients with soft or medium hearing impairment represented as HearingFunctioning would need a high sound level, while audio would be completely excluded for persons with serious impairments in order to use different interaction modalities. The *subtitle* is an enumeration parameter with values \{ *none*, *yes*, *no*\} specifying the need of supporting audio messages through text. Patients with no, soft, or medium impairment of SeeingFunctioning and medium or serious impairment of HearingFunctioning would need subtitles to better understand instructions and messages from the robot.

The *font size* is a binary parameter with values \{ *regular*, *large*\} specifying the size of the font of text messages and subtitles, if used. Patients with medium impairment of SeeingFunctioning would need *large* fonts in text messages in order to better read their content. Finally, *explanation* is a binary parameter (i.e., *yes* or *non*) specifying the need of explaining an exercise to a patient before its execution. Such instructions would be particularly needed for patients with impaired MemoryFunctioning or OrientationFunctioning. Clearly, the way such explanations are carried out complies with the interaction parameters described earlier.

## 5 Feasibility Assessment

**FIGURE 3:**
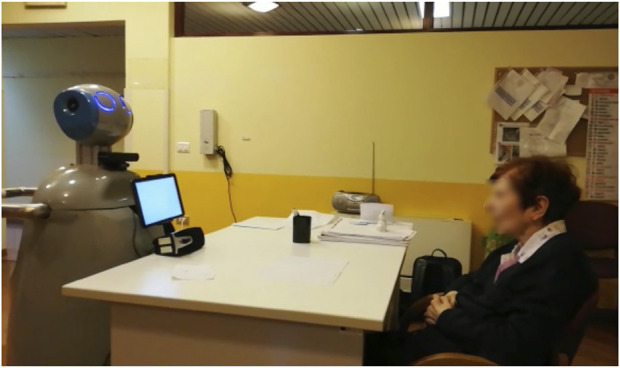
Experimental set-up: ASTRO robot is administering the MMSE test to the subject.

To demonstrate the feasibility of our cycle-based approach, we considered ASTRO, an assistive robot equipped with several sensors (i.e. laser, RGB-D camera, microphones, speakers, and force sensors) and two tablets (Fiorini et al., 2020b) (see Figure 3). We deployed on ASTRO the architecture proposed in Section 2 augmenting its capabilities with the cognitive functionalities presented in Section 2.2 in order to implement the human-in-the-loop cycle proposed in Section 2.1. Then, we demonstrated the feasibility of such robotic functionalities to support a clinician while responding to specific needs of some older adult users. In particular, eight elderly persons, 3 males and 5 females (avg. age 82.25 years old, range 72–91 years old), were enrolled for this study. All the recruited subjects live in a residential facility in the same geographical region^5^.

The ASTRO robot equipped with the new proposed functionalities was tested to demonstrate its ability to support a clinician in realizing the functionalities to 1) represent user profiles with respect to ICF (*who you are*), 2) synthesize personalized intervention plans (*what you need*) considering a set of 10 cognitive tests typically used to further investigate and evaluate the cognitive state of a person, and 3) select the appropriate interaction modalities of the robot (*how you like it*) among the ones supported by the robotic platform. An off-line analysis and discussion of the results with the clinicians are reported at the end of the section to emphasize the importance of the *human-in-the-loop* approach (see again Figure 1).

### 5.1 Demonstrating User Profiling and Profile Representation

As shown in Figure 1, user profiling is necessary at the beginning of each intervention cycle to set/update robot knowledge about the health state of an assisted person. The outcome of this step is a *profile* describing the cognitive state of a person with respect to the developed ontological model. A correct acquisition of this information is crucial for the efficacy of the synthesized intervention plan. The robot indeed relies on the *user profile* to *infer* the set of cognitive tests (i.e., *stimuli*) that are suitable for the considered user and then *decide personalized intervention plan* (i.e., the further assessment).

The user profile is generated according to the scoring obtained through the administration of the Mini-Mental State Examination (MMSE). The MMSE represents the most used screening test for cognitive status, and it is adopted worldwide by clinicians to briefly assess persons with the suspect of dementia. It encompasses 21 items which cover tests of orientation, recall, registration, naming, comprehension, calculation and attention, writing, repetition, drawing, and reading (Folstein et al., 1983). The cognitive status level is obtained by summing the score of the individual items and normalizing it based on the educational level and the age of the patient. Decreasing scores of repeated tests highlight deterioration in cognition. In particular, the participants were asked to undergo the MMSE administrated by ASTRO. The assessment was performed with a Wizard-of-Oz (WOz) method. A clinician guides the robot through the examination phases using a dedicated web interface. The patient is not aware of the presence of the clinician and he/she directly interacts with the robot. The web interface allows the clinician to select the appropriate MMSE tests to perform. The tests require different interaction modalities between the user and the robot, for example, asking questions to the user or showing images to the user through the front tablet. The clinician can ask the robot to repeat the test if necessary.

The caregiver stores the results of the assessment into the robot knowledge base through the same dedicated technical interface. The overall score of the MMSE is automatically processed by the robot at the end of the session, by parsing the annotated answers. The robot automatically correlates MMSE items with (relevant) ICF functions the robot uses to represent the cognitive state of a patient. According to this correlation, the robot then builds a user profile by mapping received MMSE scores to ICF scores. Table 1 shows how each ICF function can be described by one or multiple MMSE categories. This is an original mapping performed by a clinician between MMSE scores and ICF for generating a user profile representation. For instance, the ICF concept MemoryFunctioning is defined as specific mental functions of registering and sorting information and retrieving it as needed. This ICF function can be described by the MMSE items which cover the recalling, counting, and spelling tests. Based on the same similarity approach, the overall mapping shown in Table 1 is obtained. In order to convert the MMSE scoring of each category into the measured level of impairment of the ICF profiling, a proportional method is used. The current MMSE score (i.e., the number of tasks correctly performed) in one category is compared to the maximum MMSE score achievable in the same category and then converted into the ICF scoring. This mapping is based on an inverted scale of values. For example, if the patient correctly accomplished the requested task of MMSE items (high values of MMSE), he/she gets a lower ICF score (*no impairment*). If the patient partially accomplished the task, an intermediate value of the ICF score is assigned (*mild impairment*). If the patient did not accomplish the task, a higher value of ICF score is attributed (*hard impairment*).

**TABLE 1:** Mapping MMSE profiling to ICF profiling.

ICF function	MMSE item	Maximum score (MMSE)	Total score (MMSE)
Orientation (ORI)	Orientation in time	5	10
	Orientation in space	5	
Attention (ATT)	Counting	5	5
Memory (MEM)	Recalling	3	13
	Counting	5	
	Spelling	5	
Perceptual (PER)	Robot’s perception	-	-
High level (HIL)	Recalling	3	11
	Counting	5	
	Comprehension	3	
	Coherent interaction	-	
Language (LAN)	Naming	2	4
	Repetition	1	
	Writing	1	
Calculation (CAL)	Counting	5	5
Communication (COM)	Coherent interaction	-	1
	Incoherent interaction	-	
Speaking (SPE)	Naming	2	3
	Reception	1	
Writing (WRI)	Writing	1	1

**FIGURE 4:**
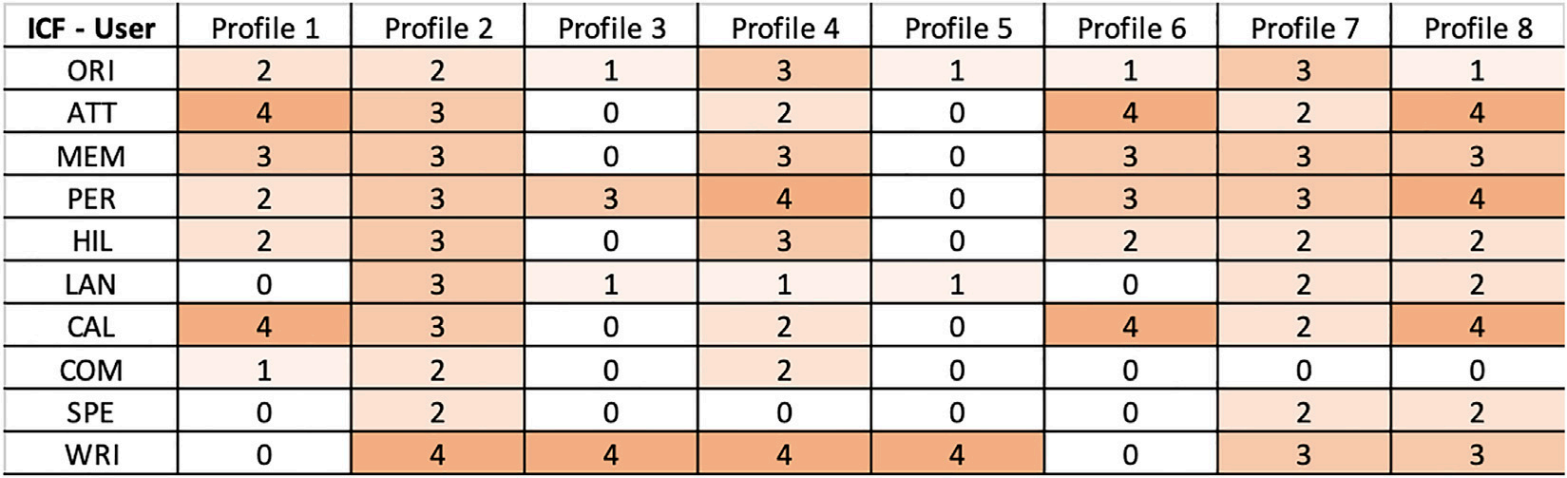
Final ICF scoring of users.

The video and audio of the administration sessions were recorded by the robot’s frontal camera. The recorded videos were off-line analyzed by the clinician to extract the quality of interaction (e.g., the number of robot’s repetition) with the robot and additional evidence of the cognitive decline (e.g., coherent and incoherent interaction) (Sorrentino et al., 2021). Table 1 reports the complete list of extracted parameters. These data are then merged with the individual MMSE score returned by the robot and manually mapped into the ICF scores, following the proposed mapping. Figure 4 reports the final results for each user. Data were then analyzed with the proposed framework and discussed with the clinician to corroborate the analysis. The clinician found an adequate representation of the profiles and validated all of them.

### 5.2 Demonstrating Intervention Personalization

The subsequent considered actions consist in the selection of a number of cognitive tests to administer, regarding the *inferred* impaired functioning qualities of a patient. The assistive capabilities of such actions depend on the features of the associated cognitive tests and thus on the functioning qualities stimulated by them. According to the inferred impairments and the recommendations generated by the developed knowledge processing mechanisms, a number of these tests are selected for administration.

We have considered a total number of 10 cognitive tests that are typically used to further investigate and evaluate the cognitive state of a person. The *Free and Cued Selective Reminding Test*, the *Rey’s Figure Test*, the *Forward Digit Test*, and the *Backward Digit Span Test* evaluate and stimulate the functioning quality MemoryFunctioning. The *Trailing Making Test form A* and the *Trailing Making Test form B* evaluate and stimulate the functioning quality AttentionFunctioning. The *Stroop Test* evaluates and stimulates the functioning quality OrientationFunctioning. The *Boston Naming Test 40-item*, the *Animals Test*, and the *Denomination Test* evaluate and stimulate the functioning quality LanguageFunctioning.

**FIGURE 5:**
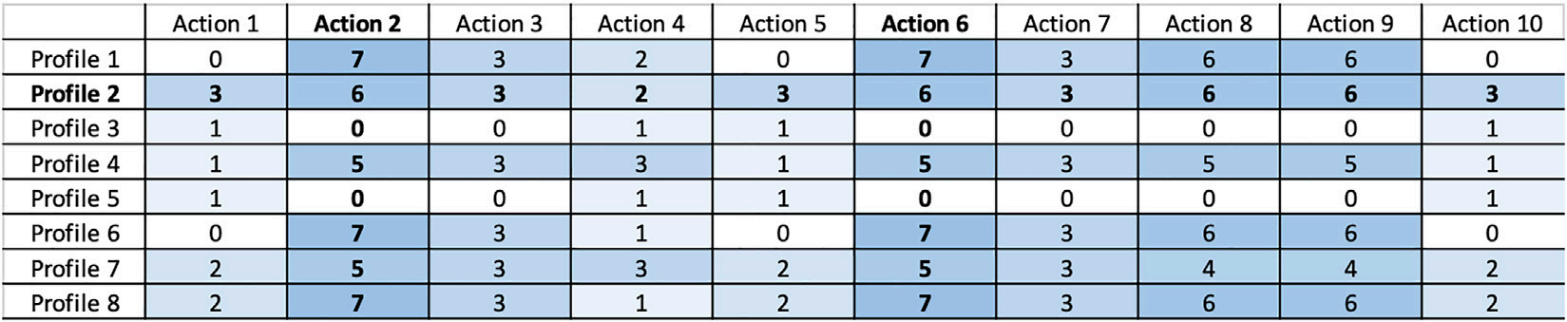
Ranking intervention actions for different profiles (action enumeration: 1) Denomination test. 2) Forward digit test. 3) Free and Cued Selective Reminding Test. 4) Stroop test. 5) Animals test. 6) Backward digit span test. 7) Rey’s figure test. 8) Trailing Making Test form B. 9) Trailing Making Test form A. 10) Boston Naming Test 40-items.

**FIGURE 6:**
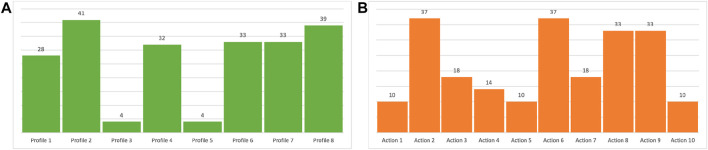
Charts show **(A)** inferred (cumulative) impairment state of patients and; **(B)** inferred impact of known actions on patients.

The experiments have been performed with the objective to demonstrate the capability of combining this knowledge with the ICF scores of Figure 4 (i.e., user profiles) to determine actions that fit the cognitive status of the profiled users and thus achieve *personalization*. Figure 5 shows results of the experiments. It specifically shows a heat-map with the *ranking values* of known cognitive tests (i.e., actions) for each user profile. The value expresses the *significance* of a specific test/action considering the cognitive impairments inferred for a particular user profile. The higher the computed ranking value the more is the relevance of a particular action for the corresponding user. Figure 6 then shows aggregated numbers pointing out the total level of impairments of the considered users in Figure 6A and the inferred impact of each action in Figure 6B. These figures clearly show the most compromised users (i.e., the users with the highest level of impairment) and the most useful actions (i.e., the actions that address the higher number and most significant impairments of users) according to robot knowledge.

The higher the ranking value the higher the “seriousness” of the associated impairments and consequently the significance of a test with respect to the cognitive state of a user. An example is *profile 8* whose cognitive state is characterized by several impairments as can be seen from the ICF scores resulting from the outcome of MMSE in Figure 4. Consequently, as shown in Figure 5, many of the considered tests have been computed as relevant to the cognitive state of this patient. Higher values have been computed for tests addressing impaired qualities, for example, the *Forward Digit Test* addressing MemoryFunctioning (medium impairment in Figure 4) or the *Trailing Making Test form A* addressing AttentionFunctioning (serious impairment in Figure 4).

Vice versa low ranking values have been computed for not so serious impairments. An example is the *user profile 5* whose cognitive state is characterized by few soft impairments (see again the ICF scores resulting from the outcome of MMSE in Figure 4). In this case, a “minimum” ranking value has been computed only for the *Denomination Test*, the *Stroop Test*, and the *Boston Naming Test 40-item* that address the soft impaired functioning qualities OrientationFunctioning and LanguageFunctioning. The outcome of the knowledge-based reasoning mechanisms has been assessed by an expert clinician. The results of this validation are reported in Section 5.4.

### 5.3 Demonstrating Interaction Personalization

Once a personalized set of interventions has been defined, user profiles are further evaluated to decide how such actions should be performed (i.e., *interaction preferences*). This reasoning step relies on a number of inference rules that link ICF scores to the interaction preferences introduced in Section 4.2. We have considered ICF scores concerning PerceptualFunction (PER) and MemoryFunctioning (MEM). MEM is linked to the interaction preference *explanation* as described in Section 4.2. PER is linked to the interaction preferences *sound level*, *font size*, and *subtitles*. As shown in Table 1, a clinician assigns a score to PER by evaluating the number of robot’s repetitions. Given the lack of a precise evaluation of hearing and seeing capabilities of users, we have used PER scores to implicitly *measure* the functioning qualities HearingFunctioning and SeeingFuncitoning and infer the related interaction preferences as described in Section 4.2.

Table 2 shows the interaction preferences inferred for the considered users. The results show the capability of the developed knowledge-reasoning mechanisms to contextualize the execution of intervention actions (and thus robot behaviors) by defining a number of coherent interaction parameters. Users with soft or no impairment conditions of PerceptualFunctioning would not require particular interaction preferences for the execution of the associated intervention actions.

**TABLE 2:** Inferred interaction parameters for different profiles.

User - Parameter	Sound level	Font size	Subtitle	Explanation
Profile 1	High	Regular	Yes	Yes
Profile 2	High	Regular	Yes	Yes
Profile 3	High	Regular	Yes	None
Profile 4	High	Large	Yes	Yes
Profile 5	Regular	Regular	None	None
Profile 6	High	Regular	Yes	Yes
Profile 7	High	Regular	Yes	Yes
Profile 8	High	Large	Yes	Yes

An example, is *profile 5* that has no impairment of PerceptualFunctioning (PER = 0 in Figure 4) and is associated to the interaction parameters ⟨ *regular*, *regular*, *none*, and *none* ⟩ in Table 2. Vice versa users with medium or serious impairment conditions of PerceptualFunctioning would require a specific configuration of robot behaviors for the execution of the associated intervention actions. Examples are *profile 4* and *profile 8* that have serious impairments of PerceptualFunctioning (PER = 4 in Figure 4) and are both associated to the interaction parameters ⟨ *high*, *large*, *yes*, and *yes* ⟩ in Table 2. These experiments show the feasibility of the developed knowledge-reasoning mechanisms in personalizing and adapting robot assistive behaviors to the health needs of different patients.

It is worth noting that the use of domain-dependent rules like the ones defined for PER would not limit the generality of the developed knowledge-reasoning approach. Rather, this situation shows how developed reasoning behaviors can be easily *tailored* to specific needs and features of different assistive scenarios.

### 5.4 Off-Line Result Discussion With the Clinician

The system identifies the subjects with a higher level of impairment, which needs more attention during a further comprehensive neuropsychological testing. Therefore, based on the stored profiles, the system suggests a broader set of further cognitive tests to the users with a higher average of cognitive issues, to provide more informative support for the clinician. We are referring particularly to subject numbers 1, 2, 4, 6, 7, and 8 that, respectively, report a raw score of 16, 17, 12, 18, 15, and 17 out 30 on MMSE. For example, subjects 1, 6, and 8 were strongly suggested to undergo the similar set of tests. In particular, those subjects, which showed a mild to medium-cognitive impairment, were asked to undergo tests related to executive functions and working memory (forward and backward digit span, and Trailing Making Test form A and B). Such cognitive impairments represent a crucial risk factor for the transition to mild cognitive impairment to a full-blown dementia syndrome. In addition, the subject number 8, which showed a more severe impairment related to languages, was suggested to undergo also a denomination test and the Boston Naming Test, both tests for language domain. Such suggestions were not mentioned in the other two subjects. Moreover, the memory function, tested by the Free and Cued Selective Reminding Test, was suggested to be studied in almost all the subjects except for the not impaired subject numbers 3 and 5.

Interestingly, the system reports less need for further test administration for subject numbers 4 and 7, respectively, the two subjects with worst cognitive performances. This is an expected performance as the implemented knowledge processing mechanisms assign ranks to intervention actions in a “non-linear way.” In particular, lower ranks are assigned to tests addressing too compromised functions as they are supposed to be managed separately. That may represent a counter-intuitive data, but a common clinic routine. Too much compromised subjects’ condition makes the clinical picture already clear and further analysis fruitless. Therefore, clinical practice is a balance between the need to accomplish an explicit vision of the case, and the economy of time and resources. On the other hand, regarding the subjects with a non-impaired neuro-cognitive profile (i.e., numbers 3 and 5. Score of 27 and 29 out 30), the system proposed fewer tests as informative. In conclusion, such results are aligned with standard clinical practice, thus the assistive robot could represent a useful tool for assessment process refinement.

## 6 Conclusion and Future Works

This study presents an original cyclic procedure to support healthcare assistance with robots endowed with a novel integration of AI-based technologies supporting *knowledge representation and reasoning* and *decision making and problem solving*, two crucial capabilities to achieve *personalization* and *adaptation* of assistive behaviors. A *human-in-the-loop* approach is pursued to define a process in which a clinician is involved into the decisional process and an interleave of cognitive state evaluation and test administration allows her to maintain the control over the decisions made by a robot and its resulting assistive behaviors. The approach was demonstrated to be feasible and effective in a realistic scenario with eight participants. A clinician supervised the procedure evaluating the robot’s behavior.

This study presented a first concrete result of a research initiative whose long-term goal is to foster the development of intelligent assistive robots capable of supporting healthcare professionals in dealing with larger number of patients. Indeed, despite the small sample size, the results suggest how the robot’s interaction parameters can be fine-tuned to the residual abilities and the cognitive profile of the person who it is interacting with. In this sense, a better understanding of patients’ social, cognitive, and biological aspects will allow assistive robots to represent such information into their cognitive system, and use it to autonomously take more initiative to support both clinicians and patients. According to the feedback obtained during the off-line discussion with the clinicians, the *decision making* module can suggest/schedule an appropriate personalized care plan. This finding can suggest that the proposed ontologies based on ICF score can be generalized to be applied to social robots to improve and personalize the human–robot interaction as well as to provide a care plan to the caregiver.

The future work plan aims at addressing two main aspects of overcoming current limitations. First, from a technical perspective, it aims to investigate user assessment and profile-building functions (e.g., via machine learning) to better identify user needs and to extend the set of assistive services supported by the cognitive architecture to enlarge the application opportunities. Second, from the user perspective, future work should consider larger involvement of participants’ cohort so as to perform a systematic evaluation to assess its concrete effectiveness, usability, and acceptance in real contexts.

## Data Availability

The original contributions presented in the study are included in the article/supplementary material; further inquiries can be directed to the corresponding author.

## Footnotes

1
https://www.who.int/standards/classifications

2
http://www.loa.istc.cnr.it/dolce/overview.html

3
https://protege.stanford.edu

4
https://jena.apache.org/index.html

5 All procedures were in accordance with the 1964 Helsinki declaration and its later amendments or comparable ethical standards
